# Does the Elapsed Time from Bleaching and the Use of Sodium Ascorbate Influence the Bond Strength of Resin Cement to Bleached Enamel?

**DOI:** 10.3390/ma16186328

**Published:** 2023-09-21

**Authors:** Ana Lílian Correia Lopes, Mara Eliane Soares Ribeiro, Jorge Henrique Pinheiro Barbosa, Max Pinto da Costa da Rocha, Mário Honorato da Silva e Souza Júnior, Sandro Cordeiro Loretto

**Affiliations:** Biomaterials Laboratory of the Postgraduate Program in Dentistry, Institute of Health Sciences, Federal University of Pará (UFPA), Belém 66075-110, PA, Brazil; analiliancl@hotmail.com (A.L.C.L.); maribe036@gmail.com (M.E.S.R.); riquepinheiro1@gmail.com (J.H.P.B.); maxpinto@globo.com (M.P.d.C.d.R.); mario.honorato50@gmail.com (M.H.d.S.e.S.J.)

**Keywords:** microshear, resin cement, antioxidant, tooth bleaching

## Abstract

The objective of this study was to evaluate different delaying times after bleaching and the use of different sodium ascorbate (SA) concentrations (10% and 35%) in the bond strength of adhesive cementation to enamel. This study used 54 sound bovine incisors, which were randomly assigned to the following groups: G1 (unbleached/control+ adhesive cementation (AC)); G2 (bleached + AC after 7 days); G3 (bleached + 10% SA + AC after 24 h); G4 (bleached + 10% SA + AC after 7 days); G5 (bleached + 35% SA + AC after 24 h); and G6 (bleached + 35% SA + AC after 7 days). After bleaching, G3, G4, G5, and G6 received an application of SA before the cylinders build up with RelyX Veneer cement. The samples were subjected to microshear test, and the results were analyzed by two-way ANOVA with Tukey’s post hoc test. Group one presented the highest mean bond strength (19.1 MPa) and G3 the lowest (16.96 MPa). There was no statistical difference between the groups tested (*p* ˃ 0.05). The fracture pattern observed was predominantly mixed. The adhesive cementation may be performed 24 h after bleaching when SA (10% or 35%) is used or after 7 days, without compromising bond strength.

## 1. Introduction

Discolored anterior teeth often cause considerable esthetic discomfort [1]. Bleaching, an inexpensive, non-invasive, rapid option for esthetic treatment, has, therefore, gained increasing popularity [2]. However, even after this esthetic treatment, complementary procedures are sometimes necessary, such as direct composite resin restorations or indirect ceramic veneer cementation [3,4].

The decreased bond strength of bleached enamel has been cited in the literature [5,6]. Nevertheless, the original values may be recovered when adhesive procedures are postponed [4,6]. A high concentration of peroxides can cause adverse effects, such as trapping high levels of oxygen in tooth structure [7]. When the restorative procedures are postponed, remineralization takes place due to saliva contact, as well as oxygen release, which may benefit the adhesion [2,6]. However, in certain situations, this delay is not possible due to restrictions, such as patient agenda or travel. Therefore, solutions to this shortcoming and alternatives to avoid postponing the adhesive restorative procedures have been sought [8]. Several studies have focused on direct restorative systems [4,5], and little data regarding adhesive cementation is available [3].

A systematic review of the use of antioxidant agents showed that regardless of type, consistency, concentration, or application type, the shear bond strength of adhesive restorative materials of bleached teeth may be recovered [4]. Sodium ascorbate (SA) is a strong antioxidant agent that is able to neutralize free reactive radicals, and when applied immediately after bleaching procedures, may recover the original (pre-bleaching) bond strength values or those observed 7 days after bleaching [4,9]. These conclusions are important, as they may shorten the whole treatment length, including eventually necessary restorative procedures [10].

In this context, some other studies [8,11,12,13] have evaluated the use of higher concentrations of SA (20–35%) to recover bond strength after bleaching procedures. The kinetic oxidant–antioxidant reaction is believed to take place faster, so the elapsed time from bleaching to restorative procedures could be significantly reduced [12]. In particular, Freire et al. [12] evaluated the efficiency of applying 35% SA to reduce the residual peroxide 40 min post-bleaching with 35% carbamide peroxide. They concluded that two applications of 1 min each were sufficient to remove these peroxides. However, no study has evaluated the effect of high concentrations of hydrogen peroxide (HP), which is normally used during in-office bleaching techniques, and the eventual interference in the adhesive cement adhesion.

Therefore, the objective of this study is to assess different sodium ascorbate (SA) concentrations (10% and 35%) and different delaying times after bleaching in the bond strength of adhesive cementation to enamel.

## 2. Materials and Methods

### 2.1. Declaration of Approval and Compliance with the Use of Research on Animal Models

This project is in accordance with Law 11.794 of 8 October 2008 and the rules issued by the National Council for Control of Animal Experimentation (CONCEA). It was approved by the Ethics Committee on Animal Use of the Federal University of Para (CEUA/UFPA), approval number 7598070119.

### 2.2. Sample Size and Specimen Preparation

The mid-parts of 54 bovine incisors were cut (10 mm high × 7 mm long) to produce the test enamel surfaces, which were mounted in PVC rings (20 mm in diameter × 1.3 mm high) with cold acrylic resin. After 24 h, the enamel surfaces were flattened on a polishing machine (AROPOL-E, Arotec, Cotia, São Paulo, Brazil) using 400- and 600-grit silicon carbide discs [6,9,11]. The sample size was calculated based on the results of a pilot study and a bond strength of 20 MPa ± 20% was adopted. GPower software version 3.1was used to analyze the data and estimate a sample of 18 cylinders per experimental group (2 cylinders were made per tooth species, totaling 18 cylinders per group). Therefore, the mounted enamel surfaces were randomly assigned into six groups (Table 1).

### 2.3. Tooth Bleaching

The mounted flattened enamel surfaces were surrounded by a photo-cured gingival barrier (Top Dam Blue, FGM, Joinville, SC, Brazil) to facilitate the application and steadiness of an even amount of 35% HP bleaching gel (Whiteness HP Blue, FGM, Joinville, SC, Brazil). Gel that was 1 mm thick was transferred to the demarcated area and checked using a periodontal probe. The bleaching gel remained in contact with the enamel for 40 min and was agitated with a microbrush applicator every 10 min to avoid bubbles. During the gel application, the specimens were placed in plastic boxes, with a small amount of water at the bottom to maintain the relative humidity, and stored in a biological oven at 37 °C. After the bleaching procedure, the specimens were removed from the oven, washed with an air–water stream for 1 min, immersed in artificial saliva, and stored again in the oven at 37 °C. These procedures were repeated three times (a total of three bleaching sessions) at intervals of 7 days. A description of the materials is given in Table 2.

### 2.4. Application of Sodium Ascorbate (10%)

The 10% SA gel was prepared in a specialized pharmacy (Fórmula & Ação, São Paulo, SP, Brazil). It was applied using microbrush points immediately after the last bleaching procedure on the teeth of the G3 and G4 groups. A thickness of 1 mm was applied and checked with a periodontal probe. The gel remained on the enamel surfaces for 10 min and was agitated with the microbrush point once per minute [5]. Then, the SA gel was washed off, and the specimens were air-dried and stored in artificial saliva.

### 2.5. Application of Sodium Ascorbate (35%)

The 35% SA gel was applied to the enamel surfaces in groups 5 and 6. For this SA concentration, the application was performed in two steps: application for 1 min, and then the SA washed off with distilled water for 1 min, and the procedure repeated; so, the antioxidant agent remained in contact with the enamel surfaces for 2 min in total [12]. Next, the gel was washed off, the teeth were air-dried, the gingival barriers were removed, and the specimens were stored in artificial saliva, as described for groups 3 and 4.

### 2.6. Testing Sample Assembly

First, the treated enamel area was delimitated using a double-faced, acid-resistant tape (Tectape, Manaus, AM, Brazil), which was perforated (0.8 mm) and had a modified Ainsworth rubber dam punch.

The delimitated area was acid-etched for 30 s using a 37% phosphoric acid gel (Condac, FGM, Joinville, SC, Brazil). After 30 s, the acid gel was washed off by air–water spray and gently air-dried. Next, the two-step Adper Single Bond Adhesive (3M ESPE, Sumaré, SP, Brazil) was rubbed on the surfaces according to the manufacturer’s instructions, air-dried, and photo-cured for 20 s (Valo, Ultradent, Indaiatuba, SP, Brazil).

The outer layer of the tape was removed, and a Tygon^®^ tube (0.8 mm internal diameter × 0.5 mm high) was positioned at the border of the delimitated area. Then, RelyX Veneer A1 cement (3M ESPE, Sumaré, SP, Brazil) was transferred to the tube lumen with a calcium hydroxide applicator and photo-cured for 40 s. Two cement resin cylinders were built up on each enamel surface. The samples were then stored in distilled water at 37 °C for 24 h.

### 2.7. Microshear Tests (MS)

The samples were then assembled on a universal testing machine (Kratos Equipaments Ltd., Cotia, SP, Brazil). A contoured stainless-steel wire (0.2 mm) was connected at one end to the load cell and at the other to the base of the cement cylinder, as close as possible to the enamel surface. Bond strength was measured by the microshear (MS) test by adjusting the test speed to 0.5 mm/min, and the values observed in the fracture were transformed to MPa.

### 2.8. Fracture Pattern Classification

After the MS tests, analysis of the fracture patterns was performed using a stereomicroscope (XTB-2B, Santo André, SP, Brazil) with 40× magnification. The fracture patterns were classified as follows: (A) adhesive—throughout the enamel–cement interface; (B) cohesive—within the enamel; (C) cohesive—within the cement; and (D) mixed—adhesive between the enamel and cement associated with cohesive within the enamel and/or cement. 

The most representative patterns of each group were selected for SEM analysis. The vestibular faces of the specimens with fracture patterns (size 1 mm × 0.5 mm) were sectioned with the aid of a double-sided diamond disc in constant refrigeration. Afterward, they were stored in silica for 24 h before being metalized and taken to the equipment. 

The images of the fractured areas were obtained by scanning electron microscopy (SEM; TESCAN, model Mira3, Tescan, LTD, Brno, Czech Republic) at 500× magnification.

### 2.9. Statistical Analysis 

Statistical analysis was performed using SPSS (version 17.0, SPSS Inc., Chicago, IL, USA). The MS bond strength results were first analyzed using the Shapiro–Wilk test to check the normality. The results were subjected to a two-way analysis of variance followed by the post hoc Tukey–Kramer test. The two factors analyzed were the antioxidant concentrations (SA 10% and SA 35%) and the time interval for adhesive cementation (24 h and 7 days). All analyses performed had a significance level set at 5%.

**Figure 1:**
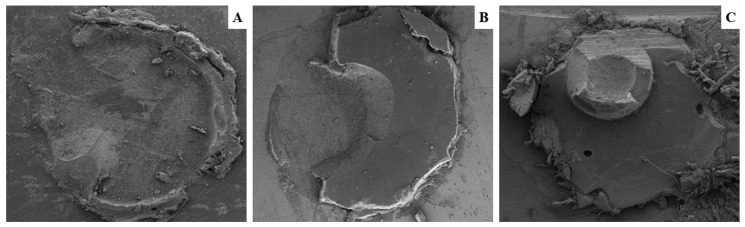
Scanning electron microscopy images (500× magnification). Adhesive type fracture (**A**), mixed type fracture (**B**), and cohesive cement fracture (**C**).

The SEM images were analyzed and used to describe the types of fractures of all specimens (Figure 1).

## 3. Results

The MS bond strength results (mean and standard deviation) are shown in Table 3. The highest mean (19.16 MPa) was observed in G1 (non-bleached control teeth) and the lowest (16.96 MPa) in G3 (bleached, SA10, and tested after 24 h).

The bidirectional analysis of variance showed no statistical difference (*p* ˂ 0.05) in the time variable or between the different treatments (negative control, SA10, and SA35). That is, the groups observed with an interval of 7 days and the groups in which AS was applied, regardless of the time interval, presented similar results.

The mean MS strengths with a 7-day delay (G2: 18.02 MPa; G4: 18.11 MPa; G6: 18.77 MPa) were higher than those observed with a 24 h delay (G3: 16.46 MPa; G5: 17.52 MPa), but no significant differences were detected (*p* > 0.05). Likewise, the mean MS strengths when 35% SA was applied (G5: 17.52 MPa; G6: 18.77 MPa) were higher than those observed with 10% SA (G3: 16.96 MPa; G4: 18.11 MPa), but there were significant differences observed (*p* > 0.05).

The descriptive analysis of the SEM images for the fracture patterns showed that the mixed type (adhesive between enamel and cement associated with cohesive within the enamel and/or cement) was predominant, regardless of the experimental group, with 78%, followed by cohesive failure (13%) and adhesive (9%).

## 4. Discussion

It is well-documented in the scientific literature that adhesive bond strength values are reduced after tooth bleaching [5,6,10,12]. Strategies are developed in an attempt to reestablish adequate values of bond strength, such as a time interval between bleaching and the adhesive procedure, as well as the use of antioxidants [6,11,14].

This study demonstrated that a delay of 7 days, or the use of SA, regardless of the concentration (10% or 35%), was able to recover a reduction in bond strength caused by whitening procedures. The average observed for the control group is comparable to the “ideal values of enamel bond strength” (20–25 MPa) mentioned in the literature [15], despite several differences in the methodology of the studies focusing on enamel bond strength. This is an important reference to position the results of the experimental groups among those that may be considered reasonable in the literature. This helps to understand that the results of the control group are acceptable to support the stresses generated before and after adhesive restorative procedures and that the use of SA, as well as the observation of at least a 7-day delay, brings the bond strength to acceptable levels as well.

The most accepted theory to explain the post-bleaching bond strength reduction is the presence of residual oxygen on the enamel surface and within its structure [15]. This may interfere with the monomer capillarity in the etched enamel, as well as the double bond conversion, compromising the polymer quality and thus the final adhesion [15,16,17]. With a 7-day delay, similar bond strength values to the control and non-bleached groups have been achieved [6,16,17]. This was confirmed through the results of this study. This phenomenon can be attributed to the time and storage medium (artificial saliva) in the procedure normally adopted in bond strength studies [6,18]. The time may allow oxygen release and the artificial saliva may be responsible for the mineral restructuring.

Although not strongly recommended, in some circumstances, the patient cannot wait for this delay, and restorative procedures should be performed immediately or after 24 h with post-bleaching treatment. Antioxidant agents have been tested in an attempt to reduce or eliminate the oxygen content of the dental substrate, recovering totally or partially the initial values of bond strength [4,5]. The acid ascorbic salt SA is a well-known antioxidant due to its capacity to reduce oxidative compounds [4]. As a low toxicity-reducing agent, it can donate high-energy electrons to eliminate free radicals due to a mechanism called passive detox [19,20,21]. There has been reported a direct relationship between the concentration of the bleaching gel and the SA [12,22]; that is, when an HP of 35% is used, a high concentration of SA (35%) must then be used to recover bond strength.

Another issue to be discussed concerns the clinical time spent performing the necessary procedures [12,13]. Because 10% SA normally demands a 10 min application time, some studies [8,11,12] have proposed the application of higher SA concentrations (20–35%) to reduce clinical time with the same effectiveness. This study used two SA concentrations (10% and 35%) at different times (10 min and 1 + 1 min). Regardless of concentration and time, the results observed for the groups that employed SA after bleaching did not present significant differences compared to the control. These results agree with those presented in a 2017 study [8], in which a two-step 35% SA application was able to recover the initial bond strength values after bleaching treatment with 35% HP. Another 2017 study [12] also demonstrated that regardless of time (60, 10, 5, 3, and 1 min) or the number of applications (one or two), 35% SA applied for 1 min was enough to eliminate all the residual peroxide and reestablish initial bond strength values.

The application of 10% SA for 10 min after bleaching procedures with low-concentration CP (carbamide peroxide) gel has been proven effective to recover bond strength before adhesive cementation [22] or direct composite resin restorations [23]. However, with high concentrations of peroxides, which are normally used for in-office bleaching, the results are somewhat controversial. In the present study, the groups that used 10% SA did not show significant differences compared to the non-bleached control group. Several studies have found similar results [24,25,26,27,28] when evaluating the bond strength of composite resins to enamel after bleaching with high-concentration gels followed by the use of low-concentration SA.

From the seventh day after bleaching, the use of SA had no influence on the bond strength results. The comparisons between groups in which SA was applied at different concentrations and times, such as group G2, did not detect statistical differences. These findings suggest that SA, regardless of concentration, should only be applied when cementation is necessary 24 h after bleaching. When postponing the adhesive cementation procedure for 7 days is possible, the use of an antioxidant agent is not necessary. The literature is very consistent in affirming the importance of postponing adhesive procedures after tooth bleaching [6,18,19,29] since oxygen before interfering in the correct polymerization of composite resins and resin cements prevents the correct formation of the hybrid layer as well as the formation of resin tags in the application of the adhesive system [17,30].

The most common fracture pattern cited in the literature [26] supports the predominant one in the present study: the mixed pattern. This kind of interface failure normally occurs when the bond strength resulting from the diffusion and in situ polymerization of monomers is higher than the substrate cohesive properties. The cohesive fracture within the substrate, which occurs in part of the bonded area, may expose one test drawback, which is caused by the incidence of non-parallel forces at the interface [31].

A limitation of this in vitro study is the use of bovine enamel, even though the literature points out that it is a suitable substrate for bond strength tests [32]. Still, more studies must be carried out, as a bleached group without the use of antioxidants for cementation in 24 h is absent in this study. An evaluation of dentin behavior in this same clinical situation must also be performed since some preparations can expose this tissue.

It is observed that the direct restorative procedures placed after bleaching have been more addressed in the literature while indirect ones, which need adhesive cementation, have not gained the same attention. Therefore, the results of the present study may contribute to a broader understanding of the subject. With a focus on short-term adhesive cementation after bleaching, the use of SA is necessary.

## 5. Conclusions

It is concluded that 10% and 35% SA before adhesive cementation may help to recover bond strength 24 h after in-office 35% HP bleaching. However, when the proposed restorative approach follows a 7-day delay, the use of an antioxidant is not necessary.

## Figures and Tables

**Table 1:** Division of experimental groups regarding the intervention to be performed.

Group	Tooth Bleaching	10% Sodium Ascorbate Application	35% Sodium Ascorbate Application	Elapsed Time from Bleaching to Cementing
G1 (negative control)	No	No	No	0 h
G2	Yes	No	No	7 days
G3	Yes	Yes (10′)	No	24 h
G4	Yes	Yes (10′)	No	7 days
G5	Yes	NO	Yes (2 × 1′)	24 h
G6	Yes	No	Yes (2 × 1′)	7 days

**Table 2:** Descriptions of the materials used, including trade names, manufacturers, and composition (according to the manufacturers).

Material	Manufacturer	Composition
Bleaching gel: Whiteness HP Blue	FGM Dental Products Ltd. Joinville, SC, Brazil	35% hydrogen peroxide, thickeners, violet inert pigment, neutralizing agents, calcium gluconate, glycol, and water
10% sodium ascorbate	Fórmula & Ação Pharmacy of Manipulation, São Paulo, SP, Brazil	Sodium ascorbate 10% and non-ionic gel qsp 120 g (water, disodium EDTA, phenoxyethanol (e) methyl isothiazolinone, aminomethylpropanol, hydroxyethyl cellulose)
35% sodium ascorbate	Fórmula & Ação Pharmacy of Manipulation, São Paulo, SP, Brazil	Sodium ascorbate 35% and non-ionic gel qsp 120 g (water, disodium EDTA, phenoxyethanol (e) methyl isothiazolinone, aminomethylpropanol, hydroxyethyl cellulose)
Dental gel conditioner Condac 37	FGM Dental Products Ltd., Joinville, SC, Brazil	37% phosphoric acid, thickener (silicon dioxide), dye (CI 61,200 blue), and deionized water
Adper Single Bond 2	3M^®^ Espe, Sumaré, SP, Brazil	Bis-GMA, HEMA, dimetacrylates, ethanol, water, an innovative photoinitiator system, and a functional copolymer of polyacrylic and polyalcenoic acid methacrylate
Light-cured resin cement: RelyX Veneer (color A1)	3M^®^ ESPE, Sumaré, SP, Brazil	An organic matrix containing Bis-GMA, TEGDMA, and zirconia/silica charge with an average particle size of 0.2–3.0 μm (approximately 47% volume)
Artificial saliva	Pharmapele, Belém, PA, Brazil	Sodium bicarbonate, 2190 mg, potassium phosphate, 1270 mg, magnesium chloride, 125 mg, calcium chloride, 441 mg, potassium chloride, 820 mg, sodium fluoride, 4.5 mg, Nipazol, 100 mg, carboxymethylcellulose, 8 mg, and distilled water, 3000 mL

**Table 3:** Mean values (±SD) of microshear bond strength (MPa).

Group	Treatment Protocol	Bond Strength (MPa) Mean (±SD)
G1	Unbleached (control)	19.16 ^a^ ± 2.48
G2	Bleached, no application of SA, cementation performed after 7 days	18.02 ^a^ ± 3.04
G3	Bleached, application of SA10 (1 × 10 min), cementation performed after 24 h	16.96 ^a^ ± 2.80
G4	Bleached, application of SA10 (1 × 10 min), cementation performed after 7 days	18.11 ^a^ ± 2.39
G5	Bleached, application of SA35 (2 × 1 min), cementation performed after 24 h	17.52 ^a^ ± 3.02
G6	Bleached, application of SA35 (2 × 1 min), cementation performed after 7 days	18.77 ^a^ ± 3.04

Equal letters indicate that there are no statistically significant differences between groups (*p* ≥ 0.05).

## Data Availability

Not applicable.
